# Living‐donor kidney transplantation for a patient with hypoparathyroidism, deafness, and renal dysplasia syndrome

**DOI:** 10.1002/iju5.12205

**Published:** 2020-07-27

**Authors:** Nobutaka Nishimura, Shunta Hori, Chihiro Omori, Makito Miyake, Satoshi Anai, Kazumasa Torimoto, Katsuya Aoki, Nobumichi Tanaka, Tatsuo Yoneda, Kiyohide Fujimoto

**Affiliations:** ^1^ Department of Urology Nara Medical University Nara Japan

**Keywords:** *GATA3* mutation, HDR syndrome, kidney transplantation, nephrocalcinosis

## Abstract

**Introduction:**

Hypoparathyroidism, sensorineural deafness, and renal dysplasia syndrome is an autosomal dominant rare genetic disease. Some patients with hypoparathyroidism, sensorineural deafness, and renal dysplasia syndrome may present with renal calcification (nephrocalcinosis) and disorder. We report the first case of living‐donor kidney transplantation for a patient with hypoparathyroidism, sensorineural deafness, and renal dysplasia syndrome.

**Case presentation:**

This case pertains to a 26‐year‐old woman who was diagnosed with congenital hypoparathyroidism 1 month after birth, following which vitamin D supplementation was initiated. In 20XX, she developed nephrocalcinosis and was confirmed to have a *GATA3* mutation; hence, she was diagnosed with hypoparathyroidism, sensorineural deafness, and renal dysplasia syndrome. In 20XX + 7, ABO‐incompatible living‐donor kidney transplantation was performed. Her renal function improved, and graft calcification was not observed.

**Conclusion:**

Over intake of vitamin D caused nephrocalcinosis. The renal function was improved after living‐donor kidney transplantation and the patient’s serum calcium levels normalized without vitamin D supplementation. Therefore, kidney transplantation should be considered a treatment option for patients with hypoparathyroidism, sensorineural deafness, and renal dysplasia syndrome.

**Abbreviations & Acronyms**
CT: computed tomography
dRTA: distal renal tubular acidosis
ESRD: end‐stage renal disease
FGF: fibroblast growth factor
FGFR1c: fibroblast growth factor receptor 1c
HDR: hypoparathyroidism, sensorineural deafness, and renal dysplasia
iPTH: intact parathyroid hormone
LDKT: living‐donor kidney transplantation
POD: postoperative day

**Keynote message**

HDR syndrome induces nephrocalcinosis, which may result in irreversible renal dysfunction. LDKT is an effective treatment option for patients with HDR syndrome.

## Introduction

HDR syndrome is a rare autosomal dominant genetic disease^1^ caused by *GATA3* mutations.^2^ Majority of patients who develop HDR syndrome have a long‐term history of calcium and/or vitamin D supplementation due to hypocalcemia caused by hypoparathyroidism; some patients also have renal and urinary tract anomalies.^3^ Excessive calcium and/or vitamin D supplementation and the abovementioned anomalies often induce renal calcification (nephrocalcinosis), which may result in renal dysfunction.^4^ To our knowledge, there are no reports of LDKT in patients with HDR syndrome.

## Case presentation

A 26‐year‐old woman had bilateral sensorineural deafness since birth and tonic‐clonic seizure 1 month after birth. Her laboratory examination showed severe hypocalcemia and hyperphosphatemia, and serum iPTH levels were undetectable. She was diagnosed with congenital hypoparathyroidism, following which oral vitamin D supplementation was initiated. In, 20XX‐5, calcification in bilateral kidneys was detected on abdominal ultrasonography, indicating renal dysfunction (serum creatinine level, 0.8–1.1 mg/dL).

**Fig. 1:**
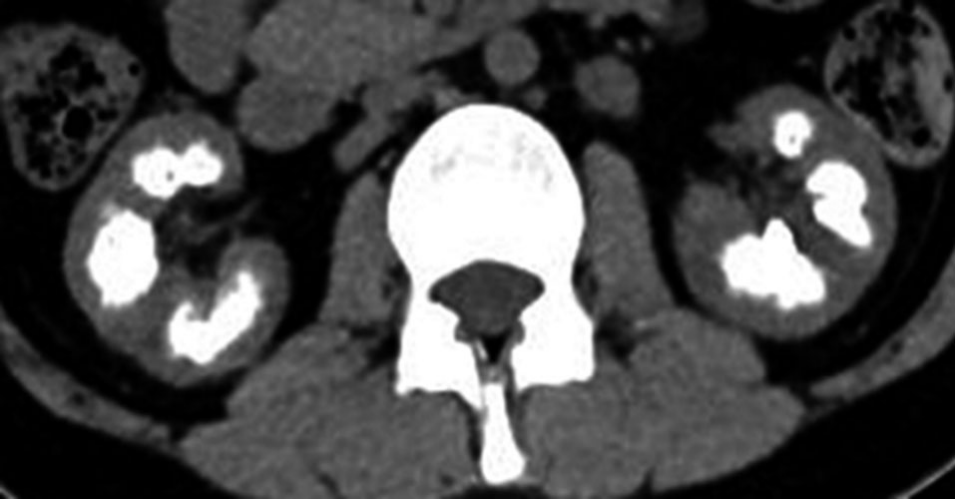
Nephrocalcinosis of native kidneys. Abdominal CT revealed severe calcification of the bilateral kidneys in October 20XX. Nephrocalcinosis was not improved although the urine calcium levels maintained a normal range thereafter. In this case, tumoral calcinosis was found only in the kidney.

Subsequently, she consulted our institute in October 20XX; the laboratory examination revealed her urine calcium/creatinine (Ca/Cr) ratio was 0.25 and serum iPTH level was 0.2 pg/mL. Abdominal CT revealed severe nephrocalcinosis (Fig. 1). Arterial blood gas analysis was unremarkable and tubular acidosis was not indicated. The urine calcium level was adjusted; her urine Ca/Cr ratio was controlled between 0.2 and 0.3. However, her renal calcification persisted. Thereafter, her renal dysfunction rapidly progressed and she was started on dialysis in February 2013, and LDKT was simultaneously planned. In June 20XX + 1, *GATA3* mutation was confirmed by genetic analysis, and she was diagnosed with HDR syndrome.

In January 20XX + 7, ABO‐incompatible LDKT was performed. The donor was her mother, who did not have *GATA3* mutation. She received double filtration plasmapheresis, plasma exchange, and immunosuppressive regimen, including cyclosporine, mycophenolate mofetil, prednisone, and basiliximab. Rituximab was also administered twice on the day and two weeks before LDKT.

**Fig. 2:**
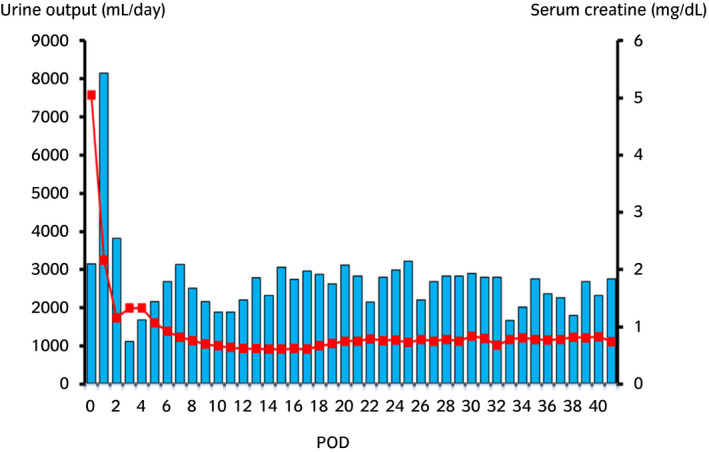
A table of serum creatinine levels and urine output after LDKT. The blue bar represents the urine output and the red line represents serum creatinine levels. Her serum creatinine level continued to decrease. It was 5.05 mg/dL before LDKT. It decreased to 2.16 mg/dL on the first day, and had maintained almost normal range since the second day after LDKT. The urine output was sufficiently produced after LDKT.

**Fig. 3:**
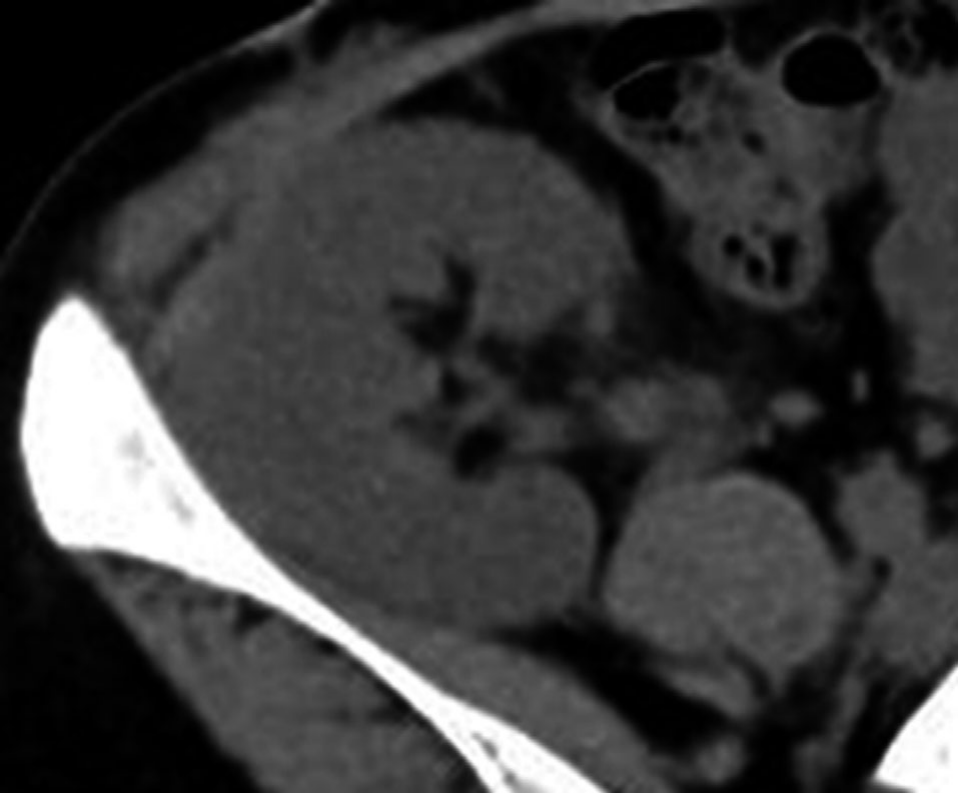
Representative image of allograft 12 months after LDKT. Abdominal CT showing that the graft had no calcification 1 year after LDKT. Urine calcium levels gradually decreased after LDKT, and graft calcification did not occur.

After LDKT, her serum creatinine level continued to decrease (Fig. 2). Her serum and urine calcium level was maintained at a normal range (urine Ca/Cr ratio ≤0.1). Her graft function was fine (serum creatinine 0.9–1.0 mg/dL), and graft calcification was not observed 1 year after LDKT (Fig. 3).

## Discussion

HDR syndrome is an autosomal dominant genetic disease characterized by the triad of hypoparathyroidism, sensorineural deafness, and renal dysplasia, which was named by Hasegawa *et al*.^1^ This is caused by a heterozygous germline mutation of the dual zinc finger transcription factor *GATA3*, which is located on 10p14‐15.^2^ *GATA3* is involved in the embryonic development of the ears, parathyroid glands, and kidneys; hence, its mutation causes triad symptoms. About 62.3% of patients with HDR syndrome have these three signs.^5^ Congenital hypoparathyroidism is clinically characterized by hypocalcemia, hyperphosphatemia, and inadequate serum iPTH levels. Intake of calcium and/or vitamin D is often indispensable soon after birth because severe hypocalcemia induces tetany, seizure, and hyperreflexia. Sensorineural deafness is often observed immediately after birth; this is bilateral and refractory. Renal dysfunction may also occur in patients with HDR syndrome because of renal and urinary tract anomalies, renal dysplasia, hypoplasia, aplasia, cyst, or vesicoureteral reflex.^3^, ^6^

Kidney transplantation is the only radical treatment for ESRD. Moreover, kidney transplantation improves survival and reduces the risk of cardiovascular events among patients with ESRD who were placed on a waiting list for transplantation as compared with long‐term dialysis.^7^, ^8^ Nephrocalcinosis is irreversible. Therefore, kidney transplantation could be an effective treatment option for patients with HDR syndrome.

One of the causes of nephrocalcinosis is dRTA.^9^ Kato *et al*. have shown that patients with HDR syndrome have congenital dRTA, which is associated with severe renal calcification.^10^ In the present case, arterial blood gas analysis revealed that there was no evidence of acidosis. On the other hand, nephrocalcinosis is associated with increasing in urine calcium.^11^ Elevated calcium levels in urine cause calcium deposition on renal tubules, leading to nephrocalcinosis. The patient had received calcium and vitamin D supplementation for 20 years after birth. Therefore, it was considered that her long‐term vitamin D supplementation augmented calcium excretion to urine and hypercalciuria caused nephrocalcinosis.

**Fig. 4:**
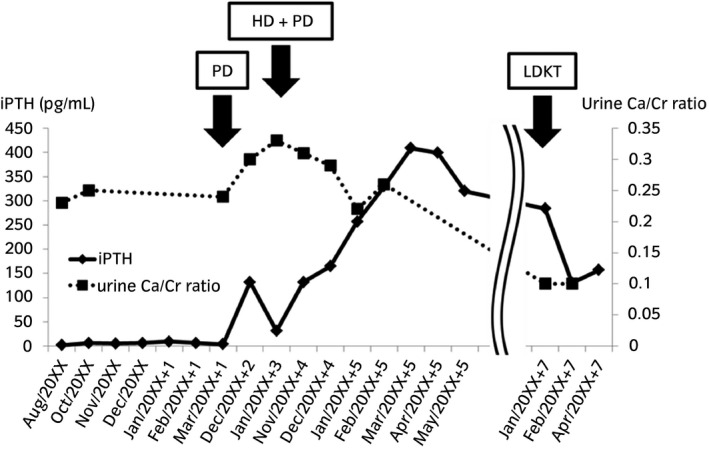
Clinical course of serum iPTH and urine Ca/Cr ratio. Her urine Ca/Cr ratio was controlled between 0.2 and 0.3 when she visited our institution. It gradually decreased to ≤0.1 after LDKT. Serum iPTH levels were originally undetectable. Since she had ESRD and dialysis treatment, serum iPTH levels were consistently >300 pg/mL, regardless of congenital hypoparathyroidism.

Since she had ESRD and dialysis treatment was introduced, her serum iPTH levels were consistently >300 pg/mL, regardless of congenital hypoparathyroidism (Fig. 4). In the general remarks, hereditary tumoral calcinosis is involved with FGF23.^12^ It binds to the receptor complex comprising Klotho and FGFR1c in osteocytes or renal proximal tubules, and has an acceleratory relevance to PTH. In chronic kidney disease, FGF23 and PTH promote the excretion of phosphate in the urine and reduce the serum phosphate level.^13^ In the present case, the function of her parathyroid glands was infinitely low due to *GATA3* mutation, but hyperphosphatemia by ESRD might intensely activate FGF23 and promote PTH secretion, leading to secondary hyperparathyroidism. After LDKT, serum iPTH levels slightly decreased with renal function improvement, but were mildly high (150–200 pg/mL); hence, tertiary hyperparathyroidism was suspected (Fig. 4). We should regularly examine serum Ca, P, iPTH. If tertiary hyperparathyroidism persists, the risk of bone‐related and cardiovascular events increases, so we consider parathyroidectomy.

It is important that the graft calcification is prevented after LDKT. We should instruct patients with HDR syndrome in dietary habits, and urged her to drink water strictly. It is also necessary to perform image examination such as ultrasonography and CT.

## Conclusion

We experienced a rare case of LDKT for ESRD due to nephrocalcinosis induced by HDR syndrome. Congenital hypoparathyroidism and ESRD induced chronic hypocalcemia, but renal function improvement after LDKT compensated for the lack of serum calcium. No evidence of graft calcification was observed 1 year after LDKT. However, long‐term observation is necessary to determine whether graft calcification occurs in the future.

## Conflict of interest

The authors declare no conflict of interest.
